# Pharmacokinetic–pharmacodynamic modeling for Moutan Cortex/Moutan Cortex charcoal and the contributions of the chemical component using support vector regression with particle swarm optimization

**DOI:** 10.1039/d0ra04111d

**Published:** 2020-06-26

**Authors:** Sixing Pan, Jianan Zhou, Sujuan Zhou, Zhangpeng Huang, Jiang Meng

**Affiliations:** College of Public Health, Guangdong Pharmaceutical University Guangzhou 510310 China 936305629@qq.com +86 020 39352095; College of Medical Information Engineering, Guangdong Pharmaceutical University Guangzhou 510006 China huangzp@gdpu.edu.cn +86 020 39352095; Medicinal Information & Real World Engineering Technology Center, Guangdong Pharmaceutical University Guangzhou 510006 China; College of Traditional Chinese Medicine, Guangdong Pharmaceutical University Guangzhou 510006 China jiangmeng666@126.com +86 020 39352169

## Abstract

Moutan Cortex (MC) and Moutan Cortex charcoal (MCC) are two kinds of Chinese medicinal materials widely used in traditional Chinese medicine (TCM) with opposite drug efficacy. And the contributions of the chemical component to the drug efficacy are still not clear. In our study, a support vector regression (SVR) model with particle swarm optimization (PSO) has been developed for simultaneously characterizing the pharmacokinetics (PK) and pharmacodynamics (PD) of MC/MCC. Then the contributions of the chemical component to the drug efficacy of MC/MCC are calculated by the weight analysis of SVR. The experimental results show that the effective substances found by the PSO-SVR model in MC and MCC are consistent with TCM theory. And the PSO-SVR model is a better model for PK–PD compared with the back-propagation neural network (BPNN). In conclusion, the PSO-SVR is a valuable tool that linked PK and PD profiles of MC/MCC with multiple components and identified the contributions of multiple therapeutic materials to the drug efficacy.

## Abstract 2

Moutan Cortex (MC) and Moutan Cortex charcoal (MCC) are two kinds of Chinese medicinal materials and effective substances are still unclear. A PK-PD model for MC/MCC is proposed using the support vector regression with particle swarm optimization.

## Graphical abstract

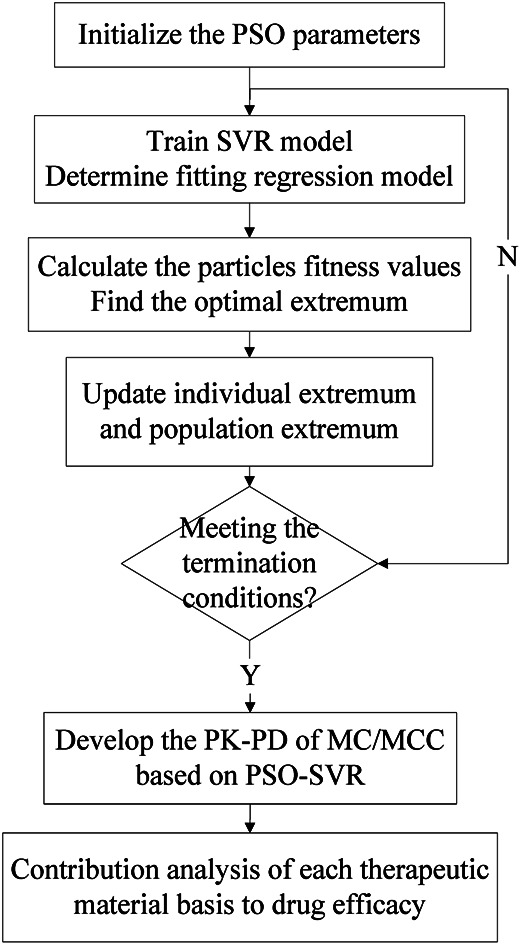

## 1. Introduction

Moutan Cortex (MC), the dried root cortex of *Paeonia suffruticosa* Andrews, is a well-known traditional Chinese medicine. According to traditional Chinese medicine (TCM) theory, MC has been commonly used for dredging meridian, removing blood stasis and eliminating inflammation from ancient times to modern China.^1^ Moutan Cortex charcoal (MCC), the processed product of MC, is produced by heating MC at a high enough temperature to turn the bark's surface black-brown. According to the TCM theory, MCC has usually been used for the treatment of gastrorrhagia, uterine bleeding and hematemesis, owing to its hemostasis and clotting effects.^2^ However, its mechanism and the contributions of the chemical component content to the drug efficacy are still not clear. The processing procedure has reduced the promoting blood circulation efficacy and strengthened the hemostasis efficacy of MCC when compared with MC. To date, although numerous researches have been conducted on processing technology of MCC, mainly focusing on the change of its physical index and chemical composition.^3–5^

For effective components study of MC/MCC, the results of the previous research indicate that benzoic acid and quercetin may be the effective substance of MC, where gallic acid and 5-hydroxymethylfurfural (5-HMF) may be the effective substance of MCC.^6^ However the mechanism in change of its PK–PD and therapeutics are still unclear.

The constitution of herbal medicine is highly complex because of the multiple components and complex structure, which leads the TCM multi-component therapeutics. Usually, the research method of traditional Chinese medicine is to obtain a single compound through systematic separation, and then conduct efficacy tests for each compound separately to clarify the pharmacodynamic substances and mechanism of action. However, this traditional method requires a lot of chemical reagents, takes a long time, has low efficiency, and fails to explain the mechanism of action of multi-component and multi-target of traditional Chinese medicine. However a pharmacological intervention based on the interaction between multiple compounds and multiple targets, to become a great technical challenge in the field of Chinese medicine.^7,8^ It is high efficiency, simple and low consumption, and need not complex chemical experiments. PK–PD model, evolving rapidly from 1960s to now with the form of mathematical models, is widely used in medical clinical treatment applications^9^ for instance exploring the therapeutic mechanism of drugs, guiding clinically rational use of drugs and providing new ideas for the optimization of clinical drug.^10–12^ Conventional PK–PD model mainly include linear models, log-linear models, Emax models and β-function models, *etc.*^13,14^. With the development of modern Chinese medicine, the PK–PD combination model is frequently used to explore the mechanism of drug efficacy in TCM.^15,16^ However, the PK–PD is a complex interrelated system, which is difficult for a simple model to clearly reflect its internal mechanism. Therefore, more advanced and accurate analysis methods are gradually used in the modern PK–PD research for instance Monte Carlo simulation models, drug-target residence time model, liquid chromatography-tandem mass spectrometry (LC-MS/MS) and artificial neural network (ANN) for the PK–PD models.^17–20^

The above models have achieved certain effects in PK–PD analysis. However due to the small number of experimental samples, the matching degree of the analysis model needs to be improved. And the new machine learning methods are urgently needed for PK–PD analysis. Support vector machine (SVM) algorithm is a machine learning method, which is more suitable for the problems of small sample, nonlinearity, over-fitting and dimension disaster when compared with others, emphasizing simultaneously on minimizing empirical and expected risks.^21–23^ The main idea of SVM is to map input space to high-dimensional feature space using kernel function, and obtains the non-linear relationship between input and output variables. The generalization ability of the model can be improved by minimizing the structure risk, and obtain good statistical results in the case of fewer input samples. The support vector regression (SVR) is a regression prediction model based on SVM, which has advantages in processing high-dimensional data. SVR has been widely used in agriculture, imaging, localization and medicinal chemistry fields.^24–26^ However, there is no unified standard for the selection of SVR parameters, which needs to be determined in accordance with specific applications.^27^

In this paper, a machine learning model based on the SVM is proposed to establish a perdition model for the change of the medicinal efficacy of MC/MCC. And a global optimization technique base on particle swarm optimization algorithm (PSO) is adopted to find the better parameters for the SVR. Based on the PSO-SVR model, the contribution weight of the MC/MCC drug concentration of its main components to its drug efficacy is analyzed. Finally, the relationships of MC/MCC drug efficacy and the contributions of main chemical component content to drug efficacy is discussed and revealed.

## 2. Materials and methods

### 2.1 Data source

All animal procedures reported in the following section have been performed according to the guide for the Care and Use of Laboratory Animals published by the National Institutes of Health (NIH Publications no. 85-23, revised 1996), approval from the Ethics Committee of Guangdong Pharmaceutical University and institutional guidelines of National Natural Science Foundation of China (NSFC). All these data were obtained from the research conducted by the Research Group of Jiang Meng supported by the Project of National Natural Science Foundation of China (no. 81473352).

Firstly, Male Wister rats, SPF grade (weighing 250 ± 30 g), were provided by Animal Experiment Center, Guangdong Academy of Medical Sciences (Certificate no. SCXK2013-0034). They have been divided into four groups, namely black group, model group (deficiency-cold and bleeding rats group), MC treated animals and MCC treated animals. Blood was collected at different time after the seventh day administration from rats, such as 0.083 0.25, 0.5, 0.75, 1, 2, 3, 4, 5, 6, 7, 10 and 12 h.

Furthermore, ten blood compounds of gallic acid, 5-HMF, 3-hydroxy-2-methyl-4-pyranone (3-H-2-M-4-P), oxpaeoniflorin, 3,8-dihydroxy-2-methylchromone (3,8-D-2-MC), paeoniflorin, benzoic acid, methyl paraben, quercetin and paeonol have been quantified using high-performance liquid chromatography method. Chromatographic conditions: Shimadzu LC-20AT system with DAD (Shimadzu Corp., Japan) was used for all analyses. Chromatographic separations were carried out at 30 °C on an Ultimate™ XB-C_18_ column (4.6 × 250 mm, 5 μm). The mobile phase consisted of acetonitrile (A) and water containing 0.2% formic acid (B). The gradient elution program was as follows: 0–10 min, 10% A; 10–20 min, 25% A; 20–45 min, 35% A; 45–80 min, 75% A; 80–90 min, 98% A; 90–95 min, 10% A. The flow rate was 1.0 mL min^−1^ and the injection volume was 20 μL. The content determination methods were all in line with the determination requirements,^6^ and the content of serum components was calculated according to the standard curve.

At the same time, pharmacodynamics were evaluated with TXB2, 6-keto-PGFlα of rats' serum were determined with enzyme-linked immunoassay detection. The details of the experiment will be published in another study.

### 2.2 Analytical method

**Fig. 1:**
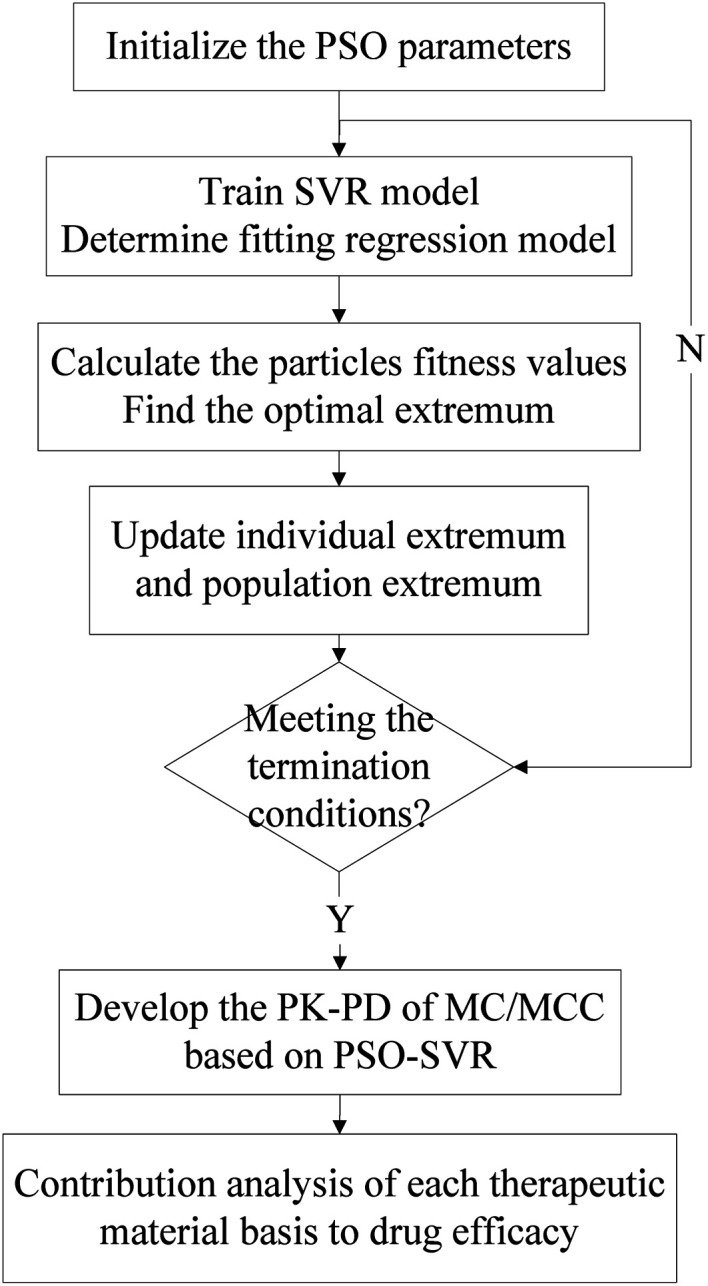
The PK–PD modeling using PSO-SVR algorithm.

The SVR model has been developed for simultaneously characterizing the PK–PD of MC/MCC. And the PSO algorithm was used to find the better parameters for the SVR. Then the contribution of therapeutic material basis was analyzed based on the PSO-SVR model. The algorithm flowchart of the PK–PD modeling using PSO-SVR algorithm is shown in Fig. 1. Firstly, the PSO parameters are initialized. Then the optimal parameters for the SVR are obtained after the PSO procedures. Then the PSO-SVR model is developed for PK–PD of MC/MCC with the optimal parameters. Finally, the contribution of each chemical component content to the drug efficacy is calculated based on the weight analysis of SVR model.

#### Construction of PSO-SVR model

Given a set of data points {(*x*_*i*_, *y*_*i*_), *i* = 1, 2,…, *N*} (*x*_*i*_ is the *n*-dimensional input vector, *y*_*i*_ is the actual test value of drug efficacy), *N* is the total number of data set. The SVR model is defined as followed:^28^

1 *f*(*x*) = *ω*^*T*^*φ*(*x*) + *b*

where *φ*(*x*) is a non-linear mapping of the input *x* and *ω* is a linear combination of *φ*(*x*) and *b* is the offset.

Using penalty factors *c* and relaxation variables *ξ*_*i*_(*i* = 1, 2,……*N*), the solution of SVR becomes an optimization problem:2
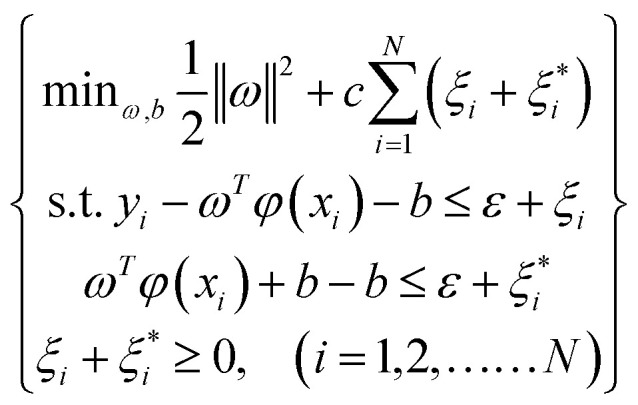


The formula (2) can be transformed by using Lagrange equation:3
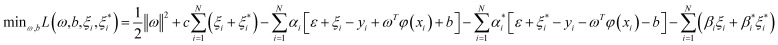
where 
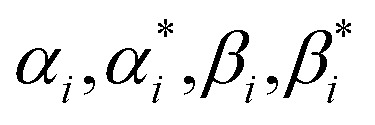
, (*i* = 1, 2,……*N*) are the multiplier of Lagrange equation. By using Lagrange's partial derivative for 
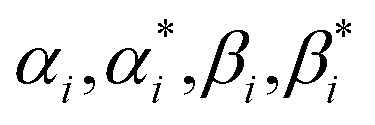
 respectively, eqn (3) can be transformed into a dual optimization problem. Finally, the decision function of SVR can be obtained:4
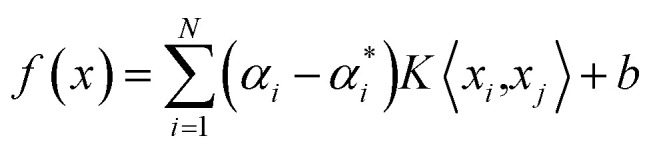
where *K*〈*x*_*i*_, *x*_*j*_〉 = *φ*(*x*_*i*_)*φ*(*x*_*j*_) is the radial basis function (RBF). And the Gaussian RBF is chosen as the RBF in our algorithm:5
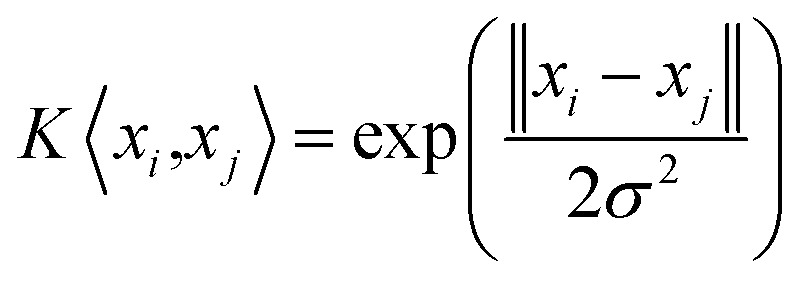


However, the penalty parameter *c* in the SVM model weighs the complexity of the model and the degree of approximation error, and the value affects the model's learning ability. And the parameter *σ* is the radial range of the RBF, which is the width of the kernel. It can be seen that the selection of the internal parameters (*c*, *σ*) of the SVR determines the prediction performance of the model. There is no standard yet to directly select the optimal value of parameter (*c*, *σ*). So the PSO algorithm is adopted to find the optimal parameter (*c*, *σ*) of the SVR.

The PSO algorithm is a parallel global search strategy based on population.^29,30^ It has faster convergence speed. It is an efficient global optimization algorithm that can effectively select the best combination of internal parameters for the SVR model and improve the prediction accuracy and generalization ability of the model. During each optimization process of PSO, the fitness function is calculated to evaluate the solution of parameter (*c*, *σ*). This function creates an output from the solution. In order to make the PK–PD model better fit the actual results, the fitness function can be specified as follows:6
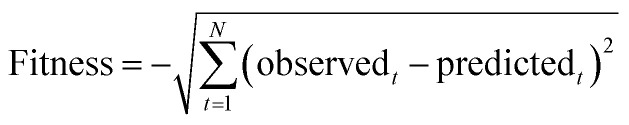
where *N* is the total number of data set. The observed_*t*_ represents the actual test value of drug efficacy and predicted_*t*_ denotes the output value of the SVR model. The smaller difference cost value means the better fitness for the model. The smaller the difference between the regression value and the actual value, the better fitness for the PK–PD model. And the optimal parameters (*c*, *σ*) are obtained after the procedures of PSO algorithm.

#### The weight analysis for of ten blood contents

Based on the PSO-SVR model, we explore the contributions of each chemical component concentration to drug efficacy. It can be seen from eqn (4) that only the corresponding training samples with non-zero coefficients can be used by the decision function, which are named as the support vectors. Therefore, the decision function can be defined as:7
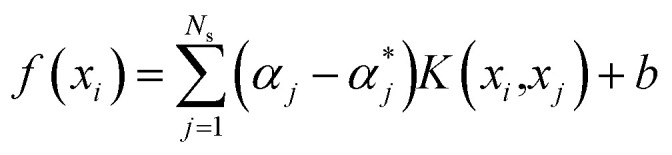
where *N*_s_ is the number of support vectors obtained after training procedures.

For the *k*th input feature, the contribution to the output can be calculated by the partial derivative:8
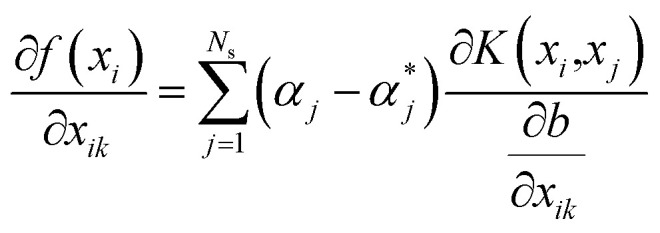


The Gaussian radial basis kernel function is selected as the kernel function in our algorithm, which is defined as:9
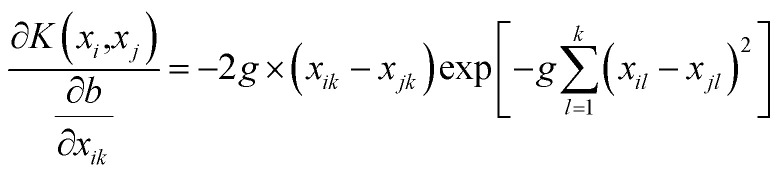
and the SVR model based on Gaussian radial basis kernel function is constructed for the PK–PD model by substituting eqn (9) into eqn (7):10
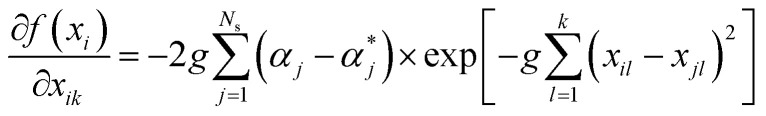


Then, the weight metric of each feature is calculated as the absolute average of the sensitivity of the output to the inputs over the total data set, which is:^31^11
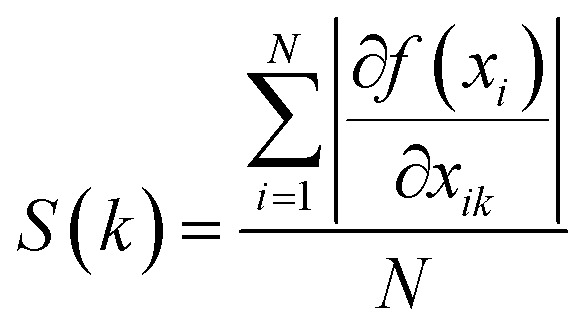


The contribution of each input feature *x*_*k*_ to the regression result *f*(*x*) is defined as:12
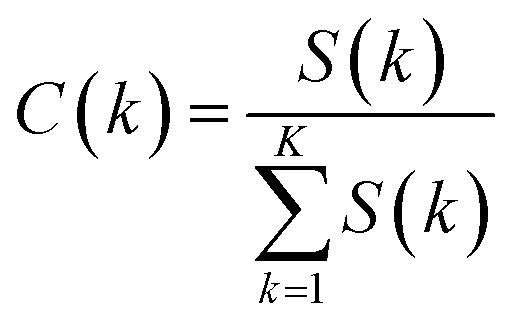


The contribution of *k*th drug concentration to the drug efficacy can be calculated by eqn (10)–(12).

## 3. Result and discussion

The SVR model is used for the PK–PD in TCM, aiming to use its methodological advantages to explore the PK–PD mechanism of MC/MCC. In our research, the blood compounds contents are used as input data, and the drug efficacy values of TXB2 and 6-keto-PGFlα are regarded as output data to trained SVR model. Then, the weights of each chemical component content to the drug efficacy are calculated by using the derivative method. The algorithm procedure is shown in Fig. 1. The proposed PSO-SVR algorithm is implemented on the MATLAB R2016a, which provides the function for SVR method. The parameters (*c*, *σ*) for the SVR method are optimized by the PSO algorithm for searching better parameters. Then the contributions of chemical component to the drug efficacy can be calculated based by eqn (10)–(12).

TXA2 is a biologically active substance released by platelet microsomal synthetic disease, which has strong vasoconstriction and platelet aggregation effects. It is because of its short half-life that, TXB2, its metabolite, is often used to reflect the metabolism level of TXA2 in the body. Prostaglandin (PGI2) is synthesized and released by vascular endothelial cells, and has anti-vasoconstriction and platelet aggregation effects. Similarly, 6-keto-PGFlα is a metabolite of PGI2, which can reflect the metabolism level of PGI2 in the body. In our study, the SVR model was developed to characterize the pharmacokinetic indexes and pharmacodynamic indexes of MC/MCC simultaneously. Then we can determine the effective substance of MC/MCC and investigate the mechanism by which processing reverses the efficacy of MC and MCC.

### 3.1 The datasets of MC/MCC

In this research, ten chemical component contents of gallic acid, 5-HMF, 3-H-2-M-4-P, oxpaeoniflorin, 3,8-D-2-MC, paeoniflorin, benzoic acid, methyl paraben, quercetin and paeonol are used as input datasets, while the corresponding drug efficacy values of TXB2 and 6-keto-PGFlα are used as output datasets. In the procedures of parameters selection, the datasets were randomly divided into training datasets and testing datasets by a ratio of five to one. Table 1 shows a group of values of chemical component content with TXB2 and 6-keto-PGFlα drug efficacy of MC determined at different time points. Table 2 has shown corresponding values of MCC.

**Table 1:** Training set of PK–PD for Moutan Cortex^a^

Time (h)	PK (content/μg mL^−1^)										PD (%)	
	Gallic acid	5-HMF	3-H-2-M-4-P	Oxpaeoniflorin	3,8-D-2-MC	Paeoniflorin	Benzoic acid	Methyl paraben	Quercetin	Paeonol	TXB2	6-Keto-PGFlα
0.08	78.06	22.89	3.18	0.96	65.06	84.19	17.98	0.69	89.32	2.97	1.12	0.05
0.25	88.72	33.84	3.67	1.21	80.75	114.71	24.20	1.61	120.70	3.35	−0.07	0.63
0.5	102.00	36.25	4.11	1.23	107.34	184.15	35.60	1.92	248.61	4.42	−0.38	0.60
0.75	133.14	51.90	2.52	1.27	120.85	75.15	39.79	3.13	256.99	6.02	0.41	0.89
1	113.30	64.61	2.15	1.46	78.55	53.40	54.21	1.43	270.83	2.20	0.89	1.26
2	96.66	119.27	1.44	1.55	37.11	53.62	51.24	0.50	301.84	4.14	−0.10	−0.53
3	73.65	103.69	1.08	1.50	36.57	35.84	65.02	0.67	412.90	4.77	0.63	−0.02
4	63.71	76.60	1.36	1.73	22.61	52.76	127.25	0.56	451.64	3.00	−0.02	1.06
6	46.25	58.06	0.75	1.02	23.00	52.06	69.82	0.60	219.12	2.11	−0.82	1.19
8	45.90	22.82	0.97	1.04	17.96	44.03	71.83	0.30	287.27	1.33	0.94	0.92
10	42.48	15.21	0.90	0.96	11.36	36.26	61.01	0.27	351.53	1.05	0.99	0.34
12	31.11	9.97	0.72	0.90	5.61	22.03	31.37	0.17	206.96	0.94	0.55	2.04

a TXB2, % = (*C*_treated animals_ − *C*_model animal_)/(*C*_model animal_ − *C*_black animal_) × 100%. 6-Keto-PGFlα, % = (*C*_treated animals_ − *C*_model animal_)/(*C*_model animal_ − *C*_black animal_) × 100%.

**Table 2:** Training set of PK–PD for Moutan Cortex charcoal^a^

Time (h)	PK (content/μg mL^−1^)										PD (%)	
	Gallic acid	5-HMF	3-H-2-M-4-P	Oxpaeoniflorin	3,8-D-2-MC	Paeoniflorin	Benzoic acid	Methyl paraben	Quercetin	Paeonol	TXB2	6-Keto-PGFlα
0.08	109.76	145.36	7.45	0.00	19.63	86.97	7.81	0.55	39.96	2.78	0.85	0.50
0.25	114.10	211.68	12.45	0.00	19.13	101.35	4.60	1.57	49.51	3.40	1.47	0.47
0.5	161.24	309.53	22.32	0.00	49.98	179.54	9.33	2.63	83.47	3.60	1.88	1.05
0.75	247.45	358.91	9.16	0.00	55.01	62.49	10.86	2.85	102.44	5.11	1.12	0.43
1	223.14	422.25	6.69	0.00	21.02	48.45	11.33	2.59	130.29	3.23	1.44	0.82
2	133.16	455.35	2.46	0.00	19.40	53.92	18.96	0.72	150.92	3.19	1.69	1.80
3	88.43	269.97	2.01	0.00	16.33	63.23	19.22	0.64	207.95	1.86	2.46	0.83
4	44.24	185.84	1.60	0.00	7.12	63.70	19.85	0.89	100.01	2.50	2.42	0.48
6	40.66	144.41	1.30	0.00	6.82	58.14	24.68	0.73	123.57	3.68	1.52	1.66
8	36.64	30.68	0.89	0.00	7.03	60.58	22.36	0.33	141.34	3.60	2.93	1.02
10	28.99	18.82	0.54	0.00	6.64	47.74	14.47	0.30	94.21	2.49	1.91	−0.60
12	27.63	6.83	0.45	0.00	4.79	15.01	9.45	0.19	62.31	2.40	0.87	4.08

a TXB2, % = (*C*_treated animals_ − *C*_model animal_)/(*C*_model animal_ − *C*_black animal_) × 100%. 6-Keto-PGFlα, % = (*C*_treated animals_ − *C*_model animal_)/(*C*_model animal_ − *C*_black animal_) × 100%.

### 3.2 Result of PSO-SVR for MC/MCC

The optimal parameter (*c*, *σ*) is obtained based on the PSO algorithm. The initial parameters of the PSO algorithm need to be set. In the proposed PSO method, the number of population and the maximum number of iterations are set as 20 and 200 separately. The parameters (*c*, *σ*) search range from 0 to 100 and the local search parameter is set as 1.5. The optimal parameter (*c*, *σ*) of the PK–PD model is shown in Table 3. For each drug efficacy, a PK–PD model is developed based on the PSO-SVR model.

**Table 3:** The optimal parameters for SVR calculated by PSO^a^

Drug efficacy values	MC		MCC	
	*c*	*σ*	*c*	*σ*
TXB2	92.721	42.244	61.188	9.858
6-Keto-PGFlα	57.641	88.907	14.526	17.69

a *c* is the penalty factors and *σ* is the radial basis function parameter.

**Fig. 2:**
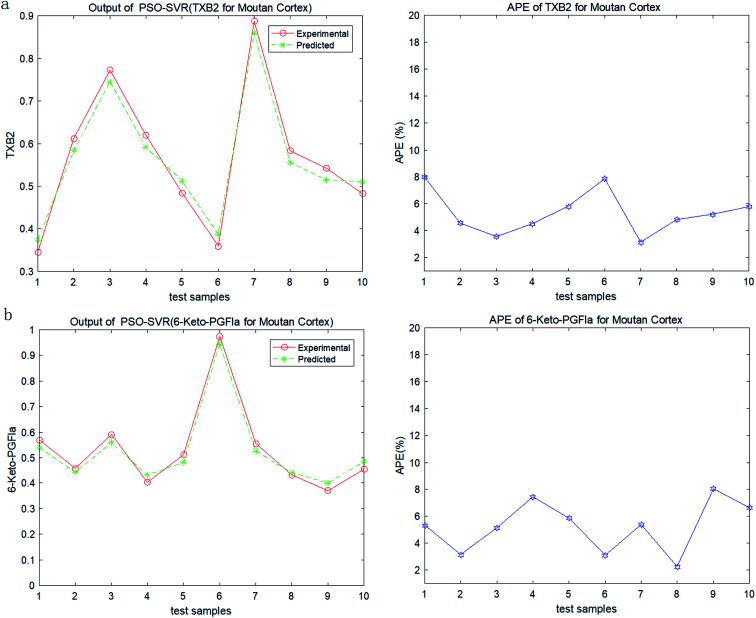
(a) Output and APE of TXB2 for MC. (b) Output and APE of 6-keto-PGFlα for MC.

**Fig. 3:**
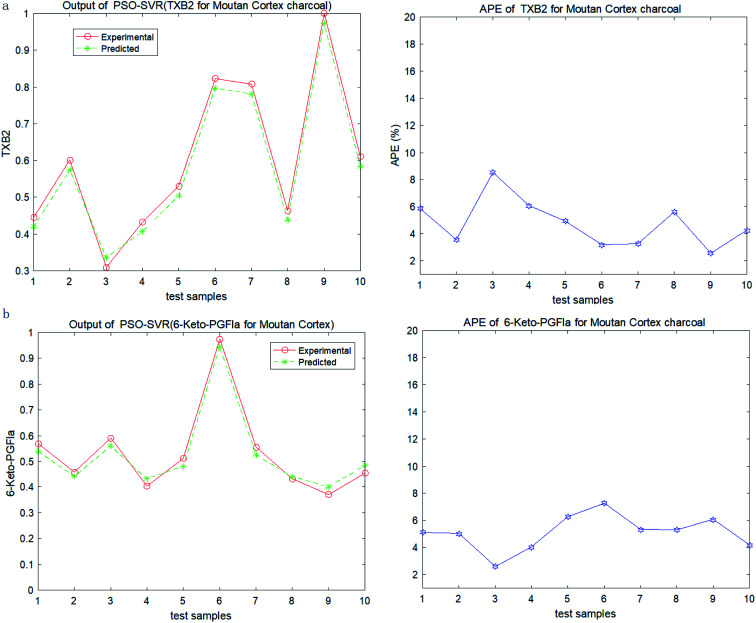
(a) Output and APE of TXB2 for MCC. (b) Output and APE of 6-keto-PGFlα for MCC.

In order to test the PSO-SVR model, ten samples are randomly selected form the datasets. And the testing results of PSO-SVR model for MC are shown in Fig. 2. The absolute percentage error (%) (APE) are calculated for TXB2 and 6-keto-PGFlα for MC separately. And the results for MCC are shown in Fig. 3. Experiment results show that all the APE values of the test samples are below 9%. The proposed PSO-SVR model better reflects the relationship between the chemical component contents and the drug efficacy.

### 3.3 Evaluation of relative contributions

According to the TCM theory, the MC has an effect of promoting blood circulation and anti-clotting; while the MCC has the effect of hemostasis and clotting. The substances of the two herbal medicine are mainly the same, but their drug efficacy of MC and MCC are opposite. In order to discover the contributions of chemical component of MC/MCC to their drug efficacy, we further explored the relationship between input data and output data of the PSO-SVR model.

The results of the contributions of chemical component contents to each drug efficacy for MC and MCC are shown in Tables 4 and 5 separately. In the PSO-SVM model for MC, it show that the contributions of benzoic acid and quercetin to 6-keto-PGFlα are positive, while the contributions of benzoic acid and quercetin to TXB2 are negative. The results suggest that benzoic acid and quercetin may be the main pharmacological substance for MC to promote blood circulation, which verify the results of our previous research. At the same time, the contributions of 5-HMF and paeoniflorin to 6-keto-PGFlα are negative, while the contribution to TXB2 are small and positive. That means the 5-HMF and paeoniflorin may be the substance for hemostasis, which needs further research.

**Table 4:** The contributions of chemical concentration to drug efficacy for MC

Components	TXB2	6-Keto-PGFlα
Gallic acid	−0.0828	−0.5801
5-HMF	***0.0624***	***−0.2874***
3-Hydroxy-2-methyl-4-pyranone	−0.0382	−0.4197
Oxpaeoniflorin	−0.0316	−0.1703
3,8-Dihydroxy-2-methylchromone	−0.1239	−0.6188
Paeoniflorin	***0.0791***	***−0.1083***
Benzoic acid	**−0.0467**	**0.2869**
Methyl paraben	−0.1357	−0.3765
Quercetin	**−0.1521**	**0.2990**
Paeonol	−0.1784	−0.4408

**Table 5:** The contributions of chemical concentration to drug efficacy for MCC

Components	TXB2	6-Keto-PGFlα
Gallic acid	**0.2592**	**−0.3165**
5-HMF	**0.1333**	**−0.0885**
3-Hydroxy-2-methyl-4-pyranone	***−0.0611***	***0.3197***
Oxpaeoniflorin	0	0
3,8-Dihydroxy-2-methylchromone	***−0.0588***	***0.0984***
Paeoniflorin	***−0.1403***	***0.3757***
Benzoic acid	−0.3633	0.2061
Methyl paraben	−0.0313	−0.0971
Quercetin	−0.2949	−0.2422
Paeonol	−0.1301	−0.0186

As shown in Table 5, the results show that the contributions of gallic acid and 5-HMF to TXB2 are positive, while the contributions to 6-keto-PGFlα are negative. These suggest that the gallic acid and 5-HMF may be the main effective substances for hemostasis in MCC, which are consistent with TCM theory. And there are the same contribution of the 5-HMF in MC and MCC.

However, the chemical components of 3-hydroxy-2-methyl-4-pyranone, 3,8-dihydroxy-2-methylchromone and paeoniflorin have positive contributions to 6-keto-PGFlα and negative contributions to TXB2. These suggest that these substances may have the effects of promoting blood circulation.

### 3.4 Compared PSO-SVR with BPNN

The SVM and artificial neural network (ANN) are two commonly used methods in artificial intelligence analysis. ANN model is a popular pattern recognition method extensively used in medical research filed due to its high degree of fault tolerance, self-organization and self-adaptive ability, low cost and time-consumption characters.^32,33^ Recently, ANN model has been used in the PK–PD analysis model of numerous medical fields for non-linear analysis.^34,35^ Back-propagation neural network (BPNN) is one of the ANNs, which has better flexibility and accuracy. The BPNN have been used for a PK–PD model for Chinese materia medica, and the experiment results are exciting.^20^ The comparison between the proposed PSO-SVR and the BPNN is implemented in the same data sets. The root mean square error (RMSE) and mean absolute percentage error (MAPE) are chosen as the evaluation indicators, which are defined as followed:13
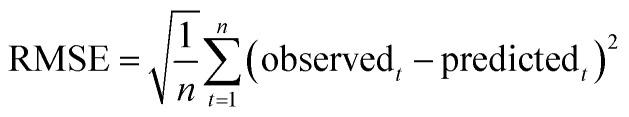
14
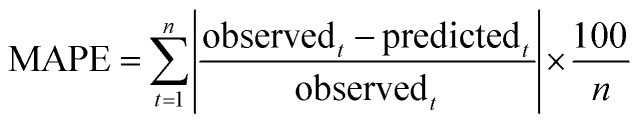


The results are shown in Table 6. The proposed PSO-SVR model for PK–PD of MC/MCC get better results, which has lower RMSE and MAPE values than the results of BPNN. Experiments show that the proposed PSO-SVR is more suitable for the simulation of PK–PD than BPNN. The reason may be that the SVR model works better in small sample datasets and the optimal parameters selection by the PSO algorithm. And experimental samples of PK–PD are always small due to the expense.

**Table 6:** Results between proposed PSO-SVR and BPNN

Drug efficacy values	MC				MCC			
	PSO-SVR		BPNN		PSO-SVR		BPNN	
	RMSE	MAPE	RMSE	MAPE	RMSE	MAPE	RMSE	MAPE
TXB2	0.031	5.31	0.069	7.82	0.029	4.65	0.57	7.74
6-Keto-PGFlα	0.042	4.86	0.076	6.24	0.036	5.26	0.642	8.67

## 4. Conclusions

In this article, a PSO-SVR model was developed to simultaneously characterize the PK–PD of MC/MCC. The results suggest that the benzoic acid and quercetin may be the main pharmacological substance for MC to promote blood circulation in MC, and the gallic acid and 5-HMF may be the main effective substances for hemostasis in MCC. These results are consistent with the TCM theory and verified by our previous research.

At the same time, there are some interesting results found by the PSO-SVR model for PK–PD of MC/MCC. The results show that the 5-HMF may have the hemostatic effect in the PK–PD model of MC, and which is verified in the PK–PD model of MCC. However, the 3-hydroxy-2-methyl-4-pyranone and 3,8-dihydroxy-2-methylchromone are found may have the effects of promoting blood circulation in PK–PD model of MCC. But no corresponding effect was found in the PK–PD model of MC. And the contribution results show that the paeoniflorin have opposite and contradictory effects in the PK–PD model of MC/MCC. All of these need more experiments to reveal the role and efficacy of these substances.

Finally we compare PSO-SVR with BPNN, which are two common methods in artificial intelligence analysis. The results show that the PSO-SVR outperform the BPNN in the PK–PD simulation of MC/MCC. The proposed PSO-SVR method provides an efficient means of modeling nonlinear relationship for PK–PD and is useful for the contribution analysis for Chinese materia medica with multi-component and multi-efficacy.

## Conflicts of interest

The authors declare no competing interests.

## Supplementary Material
