# Correction: Pharmacokinetic–pharmacodynamic modeling for Moutan Cortex/Moutan Cortex charcoal and the contributions of the chemical component using support vector regression with particle swarm optimization

**DOI:** 10.1039/d0ra90089c

**Published:** 2020-08-27

**Authors:** Sixing Pan, Jianan Zhou, Sujuan Zhou, Zhanpeng Huang, Jiang Meng

**Affiliations:** College of Public Health, Guangdong Pharmaceutical University Guangzhou 510310 China 936305629@qq.com +86 020 39352095; College of Medical Information Engineering, Guangdong Pharmaceutical University Guangzhou 510006 China huangzp@gdpu.edu.cn +86 020 39352095; Medicinal Information & Real World Engineering Technology Center, Guangdong Pharmaceutical University Guangzhou 510006 China; College of Traditional Chinese Medicine, Guangdong Pharmaceutical University Guangzhou 510006 China jiangmeng666@126.com +86 020 39352169

## Abstract

Correction for ‘Pharmacokinetic–pharmacodynamic modeling for Moutan Cortex/Moutan Cortex charcoal and the contributions of the chemical component using support vector regression with particle swarm optimization’ by Sixing Pan *et al.*, *RSC Adv.*, 2020, **10**, 24454–24462, DOI: 10.1039/D0RA04111D.

The authors regret that the name of one of the authors (Zhanpeng Huang) was shown incorrectly in the original article. The corrected author list is as shown above.

The Royal Society of Chemistry apologises for these errors and any consequent inconvenience to authors and readers.

## Supplementary Material

